# Supplementary material for: The fraternal birth-order effect as a statistical artefact: convergent evidence from probability calculus, simulated data, and multiverse meta-analysis
Source: PeerJ. 2023 Aug 18;11:e15623. doi: 10.7717/peerj.15623 (PMC10441532; doi:10.7717/peerj.15623)
Supplement: Supplemental Information 1 [file peerj-11-15623-s001.pdf]

Supplemental Materials for “The Fraternal Birth-Order Effect as a Statistical Artefact: Convergent Evidence from Probability Calculus, Simulated Data, and Multiverse Meta-Analysis”

Johannes K. Vilsmeier[1] Michael Kossmeier[1] Martin Voracek[1]

Ulrich S. Tran[1,*]

[1] Department of Cognition, Emotion, and Methods in Psychology, Faculty of Psychology, University of Vienna, Liebiggasse 5, A-1010 Vienna, Austria

* Correspondence: Ulrich S. Tran <ulrich.tran@univie.ac.at>

3/17/23

Table of contents

S1 Review of and Comments on Additional Meta-Analyses on the Fraternal Birth-Order Effect ... 2

S1.1 Jones and Blanchard (1998) ... 2

S1.2 Blanchard (2004) ... 5

S1.3 Blanchard and VanderLaan (2015) ... 8

S1.4 Blanchard, Krupp, VanderLaan, Vasey, and Zucker (2020) ... 9

S2 Explanations of Equations in Part I ... 10

S3 Converting Inequalities About Odds Ratios to Inequalities About Probabilities/Proportions ... 11

S4 Additional Researcher Degrees of Freedom in Primary Studies ... 11

S5 Simulation Study in Part II ... 13

S5.1 Methods ... 13

S5.2 Further Evaluation and Sample-Size Considerations ... 15

S5.3 Models ... 16

S5.4 Summary Plots of Models Fitted to Simulated Data ... 17

S6 Meta-Analyses in Part III ... 19

S6.1 Meta-Analyses of Specific Subsets using the Difference in Proportion ... 19

S6.2 Results of the Fixed-Effect Model Analyses ... 28

S7 Distribution of *S* Values Under the Null Model ... 31

S8 Parametric Bootstrapping Plots ... 31

S9 Specification Curve Analysis over the Difference of Male and Female Samples ... 34

References 37

S1 Review of and Comments on Additional Meta-Analyses on the Fraternal Birth-Order Effect

S1.1 Jones and Blanchard (1998)

The very first meta-analysis (Jones & Blanchard, 1998) introduced two new measures of relative birth order, the *fraternal* and *sororal indices*. The *fraternal index* (FI) can be defined as an individual's number of older brothers divided by their number of older brothers plus the number of younger brothers; i.e., the proportion of an individual's older brothers relative to the individual's total number of brothers. By analogy, the *sororal index* (SI) is equivalent to an individual's proportion of older sisters relative to their total number of sisters.

With respect to the notation introduced in the main text, the FI and SI for the ith individual are given by

$$\mathrm{FI}_i = \frac{\#\mathrm{OB}_i}{\#\mathrm{All\ Brothers}_i} \quad \text{and} \quad \mathrm{SI}_i = \frac{\#\mathrm{OS}_i}{\#\mathrm{All\ Sisters}_i}.$$

Jones and Blanchard (1998) stated that, assuming the absence of the FBOE, the mean FI and SI should be 0.5 for both homosexual and heterosexual men. Hence, under the FBOE, the mean FI in homosexual men should shift towards 1, while the mean FI in heterosexual men should shift towards 0. But the magnitude of this shift towards 0 in heterosexual men should be only a fraction of the magnitude of the shift towards 1 in homosexual men, due to homosexual men making up only approximately 2% of the male population (Cantor et al., 2002). Jones and Blanchard (1998) observed this pattern of shifts in the FIs across 872 homosexual and 2,115 heterosexual men taken from nine samples (see Table 5 in the main text for samples which were included). Such a group difference in the mean FIs would not only be expected under the FBOE, but also under a more general older sibling effect. That is, if homosexual men had a greater number of both older brothers and sisters than heterosexual men. In order to show that the difference between homosexual and heterosexual men in the mean FI is driven solely by by a greater number of older brothers, but not by a greater number of older siblings overall, one could compare the mean paired differences, *the difference-in-difference*, of FI-SI between groups. Jones and Blanchard (1998) found this difference-in-difference to be 0.036. Given the reported summary statistic of $t(3165) = 2.01$, we were able to construct an approximate 95% confidence interval CI of [.001, .072], which includes a wide range of compatible values.

The samples included in Jones and Blanchard (1998) did not correspond to the complete set of studies which had already been published by 1998 (the year of publication). At least three separate samples from two studies (Blanchard & Bogaert, 1998; Bogaert et al., 1997) were omitted. Both of these studies were co-authored by Blanchard, thus raising the question as to why they were not included in the meta-analysis.

Since then, a number of further studies (Blanchard et al., 1998; Bozkurt et al., 2015; Gómez-Gil et al., 2011; Schagen et al., 2012; Skorska et al., 2020; VanderLaan & Vasey, 2011; Vasey & VanderLaan, 2007) reporting fraternal and/or sororal indices (or some modified version thereof) have been published. However, none of these compared the mean paired difference of the fraternal and sororal indices between homosexual and heterosexual men. An update to Jones and Blanchard's meta-analysis is impeded by the non-reporting of standard deviations for FIs and SIs by Jones and Blanchard (1998), as well as the non-reporting of correlations between fraternal and sororal indices in all samples mentioned in the main text (Table 5). Beyond that, the fraternal and sororal indices once again are ratios. This complicates their statistical analysis to an unnecessary degree (see our discussion about the use of ratios in the main text in Part I).

Here, we also present *t* tests for both the between-group difference in the mean FI and the difference in the mean FI-SI for each of the 18,000 replicates in the simulation study of the main text in Part II. As can be seen in Figure S1, the FI behaved just like the MROB, the MPOB, the OBOR, and the other models and measures, which do not adjust for the number of older siblings (see Figure 2 in the main text). Used as a criterion for the existence of the FBOE, the mean FI would bring about false-positive decisions at a much greater frequency (close to 100% for some conditions) than expected with a significance level of 5%.

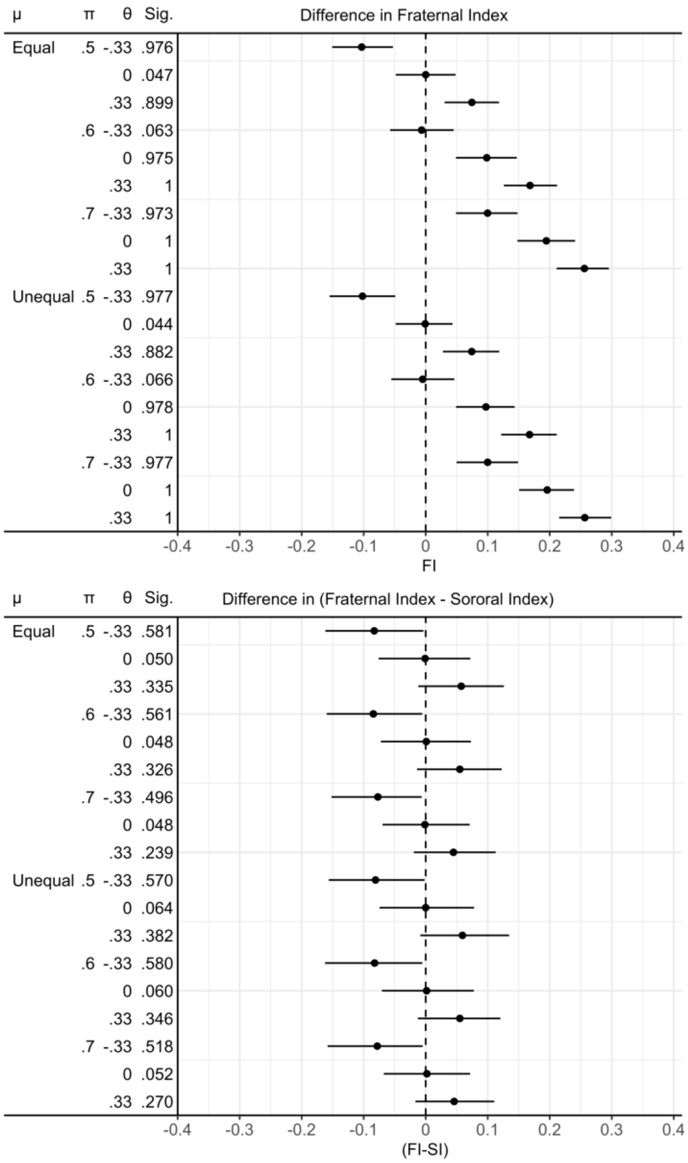

Figure S1. Results of the difference between the fraternal index (FI; top) and the paired difference of fraternal and sororal indices (FI-SI; bottom) across all 18,000 simulations of homosexual and heterosexual participants (see main text for the description of the simulation in Part II). Points depict the means of the differences (horizontal axis). The endpoints of the error bars depict the .025 and .975 quantiles of the distributions of differences. The column Sig. indicates the proportion of differences with $p < .05$ (two-tailed).

S1.2 Blanchard (2004)

The second meta-analysis (Blanchard, 2004) contained five additional samples (see Table 5 in the main text). Instead of contrasting FIs and SIs (Jones & Blanchard, 1998), Blanchard (2004) treated the samples as equally weighted single observations and used the mean number of older brothers, older sisters, younger brothers, and younger sisters in each sample as predictor variables in a logistic regression model, with the sexual orientation of the groups as the outcome variable. The *p* value of the change in the logistic regression's deviance statistic associated with the removal of each predictor separately was used for assessing the effect of each predictor. Only the removal of the mean number of older brothers from the model yielded a statistically significant change in the model's deviance. Hence, Blanchard (2004) concluded that the number of older brothers must have increased the odds of homosexual orientation. Blanchard (2004) reported neither regression coefficients nor shared the data used in this meta-analysis. We reconstructed Blanchard's (2004) analysis using the data of the 14 samples in (Blanchard, 2004) as reported in the appendix of the third meta-analysis (Blanchard, 2018a).

The file `blanchard_2018_append.csv` contains the total number of older brothers, older sisters, younger sisters, and younger brothers for the 30 samples as reported by in the third meta-analysis (Blanchard, 2018a). Note that the data reported by Blanchard (2018a) are slightly different from the data we used in our meta-analyses in the main text, as we excluded bisexual individuals whenever possible.

First, we preprocessed the data by selecting the subset of samples included in Blanchard (2004) and computing the corresponding group means. Samples included in Blanchard (2004) are indicated by `1`s in the column named `inB`.

```
library(tidyverse)
# Load Data
# sibship data as reported by Blanchard (2018a)
blanchard_2018 <- read_csv('../Data/blanchard_2018_append.csv')

# Select subset of samples included in Blanchard's 2004 meta analysis
# inB denotes whether sample
blanchard_2004 <- blanchard_2018[blanchard_2018$inB==1, ]

# compute sample means

blanchard_2004 <- blanchard_2004 %>%
  mutate(
    ob_avg = ob/n,
    os_avg = os/n,
    yb_avg = yb/n,
    ys_avg = ys/n
  )

#recode variable "group" to 0 = heterosexual 1 = homosexual
blanchard_2004$group <- as.factor(ifelse(blanchard_2004$group == 'Homosexual'
, 1,0))
```

The data for reconstructing the second meta-analysis are listed in Table S1.

Table S1. *Data for reconstructing analyses by Blanchard (2004).*

study	group	ob_avg	os_avg	yb_avg	ys_avg
Blanchard & Bogaert (1996a)	1	0.6959	0.5882	0.5031	0.4831
	0	0.5839	0.539	0.5779	0.5758
Blanchard & Bogaert (1996b)	1	0.7053	0.6026	0.5397	0.5861
	0	0.4816	0.4747	0.6613	0.6336
Blanchard & Bogaert (1998), OA	1	0.8269	0.7115	0.4615	0.5705
	0	0.8902	0.9133	1.069	0.9422
Blanchard & Bogaert (1998), OP	1	1.145	1.13	0.7101	0.8116
	0	1.165	0.937	1.386	1.189
Blanchard & Bogaert (1998), OC	1	1	0.9524	0.8571	0.619
	0	1.077	1.091	1.028	1.084
Blanchard et al. (2000)	1	1.077	0.7846	0.8154	0.6615
	0	0.7566	0.6908	0.8421	0.8092
Blanchard & Sheridan (1992)	1	1.041	0.8187	0.7876	0.5803
	0	0.4908	0.4762	0.7399	0.5751
Blanchard & Zucker (1994)	1	0.5026	0.4499	0.4903	0.4974
	0	0.4377	0.3559	0.516	0.5694
Blanchard et al. (1995)	1	0.6346	0.4295	0.3141	0.25
	0	0.4167	0.391	0.4167	0.4103
Blanchard et al. (1996)	1	0.9904	0.6346	0.5577	0.4615
	0	0.6456	0.3924	0.7215	0.7089
Blanchard et al. (1998)	1	0.5325	0.426	0.4805	0.4519
	0	0.3244	0.4267	0.4178	0.4178
Bogaert et al. (1997)	1	0.7647	0.8088	0.4853	0.6176
	0	0.5614	0.7018	0.8772	1.035
Ellis & Blanchard (2001)	1	0.6686	0.4857	0.4914	0.4914
	0	0.5088	0.4964	0.4985	0.4449
Zucker & Blanchard (1994)	1	0.449	0.4898	0.3469	0.2857
	0	0.3929	0.3452	0.3571	0.3214

Notes. Column group indicates the sexual orientation of a sample (0 = heterosexual, 1 = homosexual), which are regarded as the subjects of analysis here; OA = "offenders against adults", OP = "offenders against pubescents", OC = "offenders against children".

We next fitted the same models as described by Blanchard (2004). First, the baseline model predicting the samples' sexual orientation from the mean number of older brothers, older sisters, younger brothers, and younger sisters. Notice that *R* returns a warning, indicating that some fitted values are 0 or 1.

```
mod_base <- glm(
  group~ob_avg+os_avg+yb_avg+ys_avg,
  family = binomial(link = "logit"),
  data = blanchard_2004
)

Warning: glm.fit: fitted probabilities numerically 0 or 1 occurred
```

We ignore this issue for now, since our goal was to reproduce the analyses. Next, Blanchard (2004) defined a second model omitting the mean number of older brothers from the list of predictors and compared this second model to the baseline model using the *p* value associated with the change in the deviance from the baseline model.

```
mod_ob <- glm(
  group ~ os_avg+yb_avg+ys_avg,
  family = binomial(link = 'logit'),
  data = blanchard_2004
)

#Compare models
anova(mod_ob, mod_base, test = 'Chisq')

Analysis of Deviance Table

Model 1: group ~ os_avg + yb_avg + ys_avg
Model 2: group ~ ob_avg + os_avg + yb_avg + ys_avg
  Resid. Df Resid. Dev Df Deviance  Pr(>Chi)
1        24     17.391
2        23      5.617  1   11.774 0.0006007 ***
---
Signif. codes:  0 '***' 0.001 '**' 0.01 '*' 0.05 '.' 0.1 ' ' 1
```

The difference between the two models' deviance statistics is almost identical to that reported by Blanchard (2004). Here, we observe a value of 11.77, Blanchard reported a value of 11.88. Blanchard (2004) did not report regression coefficients for any of the models. An analysis of deviance reveals very little about the size and direction of the association between outcome and predictors. The regression coefficients for the baseline model were:

```
summary(mod_base)


Call:
glm(formula = group ~ ob_avg + os_avg + yb_avg + ys_avg, family = binomial(li
nk = "logit"),
    data = blanchard_2004)
```

```
Deviance Residuals:
     Min        1Q    Median        3Q       Max
-1.37569  -0.00906   0.00000   0.00003   1.67358

Coefficients:
            Estimate Std. Error z value Pr(>|z|)
(Intercept)   -2.240      3.973  -0.564    0.573
ob_avg       141.864    137.833   1.029    0.303
os_avg       -42.664     51.456  -0.829    0.407
yb_avg      -133.047    134.924  -0.986    0.324
ys_avg        28.649     45.099   0.635    0.525

(Dispersion parameter for binomial family taken to be 1)

    Null deviance: 38.816  on 27  degrees of freedom
Residual deviance:  5.617  on 23  degrees of freedom
AIC: 15.617

Number of Fisher Scoring iterations: 11
```

According to the above results, the estimated regression coefficient associated with the mean number of older brothers (ob_avg) is 142 lnORs, which corresponds to an OR of 4.08×10^{61}. An estimate this large clearly indicates that entire analysis cannot be trusted.

S1.3 Blanchard and VanderLaan (2015)

In the third meta-analysis, Blanchard and Vanderlaan (2015) aggregated 14 samples, which were neither collected nor analysed by themselves or any of their collaborators. The study was described as a "simple meta-analysis that would be as free from our own potential unconscious biases as possible" (Blanchard & VanderLaan, 2015, p. 1506). Yet, this meta-analysis excluded the sample from a national cohort study with more than 2 million participants (Frisch & Hviid, 2006), which assessed sexual orientation via the registered sex of a person's spouse (i.e., used same-sex marriage as an indicator of homosexual orientation). Notably, said study (Frisch & Hviid, 2006) concluded to have found no evidence for a greater number of older brothers in homosexual men, compared to heterosexual men.

Blanchard and VanderLaan (2015) reported a positive sign for 13 out of the 14 comparisons between homosexual and heterosexual men in the included studies, meaning that the average number of older brothers was greater for homosexual than heterosexual men. Testing this number against the null hypothesis of an equal probability of positive and negative signs for these comparisons yielded a p-value of .002, which is well below the significance threshold of .05. For the respective average number of older sisters, only 10 out of 13 comparisons had a positive sign (only 13 samples reported the mean number of older sisters). This number was not statistically significant, $p = .097$. Based on this pattern of statistically significant and statistically not significant effects, Blanchard and VanderLaan (2015) concluded that homosexual men, on average, had more older brothers than heterosexual men, whereas no such difference existed with respect to the average number

of older sisters. However, the lack of statistical significance does not imply the absence of an (even similar-sized) effect with respect to older sisters here. It is yet another example of the "the-difference-between-significant-and-not-significant-is-not-itself-statistically-significant"-fallacy (Gelman & Stern, 2006). Observing that one effect is significantly different from the point-null effect and that a second effect is not significantly different from the point-null effect, does not necessarily indicate that the two effects are themselves significantly different from each other. If anything, Blanchard and VanderLaan (2015) provided evidence for a more general older sibling effect (i.e., homosexual men have more older brothers and sisters than heterosexual men; the exact same point had already been raised by Gelman and Stern, 2006, who used the analysis in Blanchard and Bogaert, 1996a, to illustrate this fallacy). Even if Blanchard and VanderLaan's interpretation of the sign test had been correct, their analysis would have provided no information about the magnitude of the supposed greate number of older brothers in homosexual men. Furthermore, the application of a sign test implies that each study should be treated as containing the same amount of information (i.e., each study carries the same weight), an assumption which cannot be justified when aggregating over differently-sized studies (Hedges & Olkin, 1985). A properly weighted meta-analysis could have led to a vastly different conclusion.

S1.4 Blanchard, Krupp, VanderLaan, Vasey, and Zucker (2020)

For the sixth meta-analysis, Blanchard et al. (2020) put forth yet another measure for quantifying the supposed greater number of older brothers, originally proposed by Khovanova (2020). The authors' implementation of Khovanova's suggestion may be described in two steps. First, the sampling space is restricted to men from two-son sibships (i.e., these men reported either a single older brother or a single younger brother and any number of sisters). Second, the following odds ratio is computed and subjected to the usual inferential machinery:

$$OR_{21} = \frac{\#\text{Second}_{\text{Hom}}/\#\text{First}_{\text{Hom}}}{\#\text{Second}_{\text{Het}}/\#\text{First}_{\text{Het}}},$$

where #Second and #First, refer to the number of second- and first-born sons. We refer to this OR as the *second-to-first-born OR*. Over a set of 14 samples, the authors reported a mean OR_{21} of 1.38. Thus, there seemed to be an excess of second-born sons among homosexual men, who have exactly one brother but any number of sisters. This was in turn interpreted as evidence for the FBOE. Again, this result carries little information about the FBOE, as it fails to account for the number of older sisters (i.e., the older sibling effect).

Moreover, and in line with Parts I and II of the main text, the second-to-first-born OR cannot distinguish between a genuine older brother effect and a more general older sibling effect. This can be seen clearly from the results obtained by fitting the OR_{21} to the simulated data reported in the main text (Figure S2; See main text for the description of simulation study in Part II).

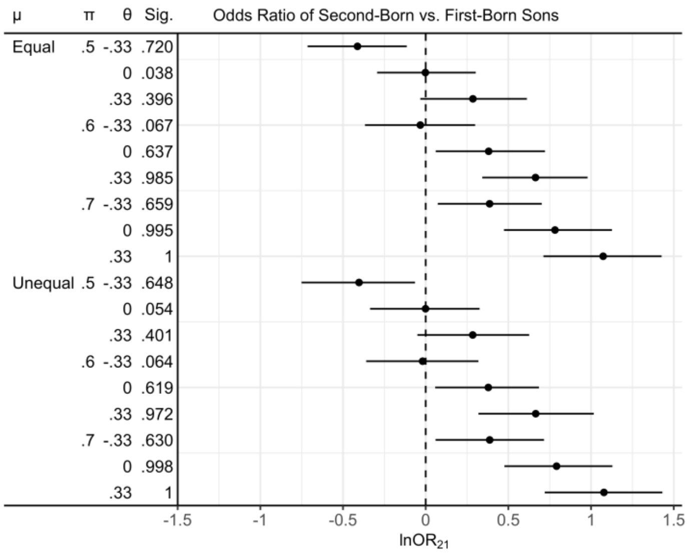

Figure S2. Results of the second-to-first-born odds ratio across all 18,000 simulations of homosexual and heterosexual participants (see main text for the description of the simulation in Part II). Points depict the average across all 18,000 point estimates (horizontal axis). The endpoints of the error bars depict the .025 and .975 quantiles of the distributions of the point estimates. The column Sig. indicates the proportion of point estimates with $p < .05$ (two-tailed).

S2 Explanations of Equations in Part I

Equation 1 states that if all of the siblings reported by homosexual and heterosexual participants are treated as the sample, the OBOR is given by the odds of observing an older brother among all siblings of homosexual individuals divided by the odds of observing an older brother among all siblings of heterosexual individuals. Per definition (e.g., Blitzstein & Hwang, 2019, p. 53), the odds of observing an older brother in either group are given by the probability of observing an older brother among all siblings divided by the probability of not observing an older brother among all siblings (i.e., the probability of observing an older sisters, a younger brother or a younger sister).

Equation 2 is just an algebraic transformation of equation 1 (see Supplement S3 for a derivation). It states that if the odds of observing an older brother among all of the siblings of homosexual individuals are greater than those same odds for heterosexual individuals, then the probability of observing an older brother among the siblings of homosexual individuals must also be greater than the same probability for heterosexual individuals, and vice versa.

The equation *in-between equation 2 and equation 3* (has no number) states that in addition to classifying the siblings in both groups (the homosexual and the heterosexual group) into whether they are older brothers or not, we can also classify them according to whether they are older siblings or not. That is, among the siblings reported by the homosexual and heterosexual participants, respecitvely, we can further distinguish between siblings who

are older and siblings who are younger than the participant. All older brothers can be found among the group of older siblings, whereas it is impossible for an older brother to be among the younger siblings.

Equation 3 states that, since there are no older brothers among the younger siblings reported by the homosexual and heterosexual individuals, respectively, the probability of observing an older brother among younger siblings is 0. Thus, the probability of observing an older brother among all siblings of homosexual and heterosexual individuals, respectively, can be decomposed into the probability of observing an older brother among the older siblings multiplied by the probability of observing an older sibling in either group (homosexual or heterosexual) in the first place.

Equation 4 combines the statements from equations 1 to 3. The inequality in the bottom row shows that one can have an *OBOR* > 1 even if the proportion of older brothers is smaller in the homosexual group as compared to the heterosexual group if the proportion of older siblings (i.e., brothers and sisters) is sufficiently greater in the homosexual group as opposed to the heterosexual group.

S3 Converting Inequalities About Odds Ratios to Inequalities About Probabilities/Proportions

Let A, B, C be disjunct events defined on a sample space. The conditional probabilities of event A given either B or C are denoted by $P(A|B)$ and $P(A|C)$, respectively. Further, let $0 < P(A|B) < 1$ and $0 < P(A|C) < 1$. By definition (e.g., Blitzstein & Hwang, 2019, p. 53) the odds of the event A (conditional on B or C) are given by $\text{Odds}(A|B) = P(A|B)/[1 - P(A|B)]$ and $OR\text{Odds}(A|C) = P(A|C)/[1 - P(A|C)]$. Then the Odds Ratio is given $\frac{\text{Odds}(A|B)}{\text{Odds}(A|C)}$.

And $OR > 1$ can be rewritten as:

$$\text{Odds}(A|B) > \text{Odds}(A|C)$$

$$\Leftrightarrow \frac{P(A|B)}{1 - P(A|B)} > \frac{P(A|C)}{1 - P(A|C)}$$

$$\Leftrightarrow P(A|B)[1 - P(A|C)] > P(A|C)[1 - P(A|B)]$$

$$\Leftrightarrow P(A|B) - P(A|B)P(A|C) > P(A|C) - P(A|B)P(A|C)$$

$$\Leftrightarrow P(A|B) > P(A|C)$$

Substituting A, B, C by OB, Hom, Het in equation 1 of the main text, equation 2 of the main text follows immediately.

S4 Additional Researcher Degrees of Freedom in Primary Studies

Having a number of different measures in one's statistical toolbox is by far not the only degree of freedom available to researchers in the field (Gelman & Loken, 2013; Simmons et

al., 2011). It is not difficult to obtain a statistically significant result under these conditions (Gelman & Loken, 2013; Simmons et al., 2011). Importantly, it does not even have to be the case that researchers try out various analysis plans (cf. Blanchard, 2018b) and only report results that turned out to be statistically significant. All that is required is a set of different hypothetically justifiable analysis plans from which researchers can choose in light of the data. Two additional examples of inconsistent data analytic decisions which have been made in primary studies on the FBOE may clarify this point.

Some studies considered a moderating effect of handedness on the excess of older brothers (Blanchard, 2008; Blanchard et al., 2006; Skorska et al., 2020), while others did not even mention handedness. Thus, when analysing data, the theoretical hypothesis of the fraternal birth order effect could be mapped at least onto two different statistical hypothesis tests: one test that does not include handedness as a moderator and another test that does. If the test which does not consider handedness turns out to be statistically significant, then this would be interpreted as supporting the theoretical hypothesis of the FBOE. If the statistical test turns out significant in only one handedness-group but not in the other, then this result could also be reconciled with the FBOE (Blanchard, 2008; not to mention the large number of ways, which may be employed for classifying handedness).

Having found a statistically significant effect, one can *post-hoc* always draw on biological theory or evolutionary psychology and put forth a potent and coherent explanation as to why an effect may be present in one handedness group, but not the other. For instance, the presence of an effect in right-handers, but not left-handers, could be ascribed to differences in the level of testosterone in utero between left- and right-handed individuals. Yet, such an approach is not identical to, and differs in significant ways from, using statistical tests for pre-experimentally derived hypotheses, employing pre-registered analysis plans that make the substantive case that the hypothesis has actually been derived beforehand.

As a second example, some studies either excluded a large number of participants (Blanchard & Lippa, 2007) or explained away not significant findings by supposing that a so called “stopping rule” obfuscated the effect of the FBOE (Zucker et al., 2007). A stopping rule entails that, due to societal or environmental factors, parents cease to reproduce; for instance, after a certain number of offspring or combination of offspring-sexes is obtained (Blanchard & Lippa, 2007). It is argued that if such a stopping rule is at play, the FBOE might not show as there are not enough younger brothers who are homosexual to detect the effect (Blanchard & Lippa, 2007).

Both the moderating effect of handedness and the presence of a stopping rule are plausible explanations. However, the inconsistency with which these covariates are included throughout the entire field, the fact that many of these explanations have been put forth post-hoc, and that there are studies that have made adjustments to their analysis plans after the primary analyses did not return a statistically significant result (Blanchard et al., 1995, 1996; Blanchard & Lippa, 2007) leave the impression that many of the decisions pertaining to the mapping of the theoretical hypothesis of the FBOE and the implied excess of older brothers in homosexual men onto statistical hypotheses were at least data driven if not motivated by a desire to obtain statistically significant results.

S5 Simulation Study in Part II

S5.1 Methods

We used the *R* packages *simstudy* (version 0.2.1; Goldfeld & Wujciak-Jens, 2021), *doRNG* (version 1.8.2; Gaujoux, 2021), *doParallel* (version 1.0.16; Microsoft Corporation & Weston, 2021a), and *foreach* (version 1.5.1; Microsoft Corporation & Weston, 2021b) to generate random draws from a four-dimensional Poisson distribution with a specified correlation matrix, wherein each draw represented a simulated participant. The four dimensions served as the variables #OB, #OS, #YB, and #YS. We chose the Poisson distribution over other discrete distributions because it provides a simple way of generating count data. Other distributions may well provide better approximations to real-world sibling data; however, we are not trying to approximate or model the distribution of sibling counts as they are encountered in real data. Rather, we aim to demonstrate the problems that emerge when applying inadequate methods and decision rules to the kind of count data inevitably encountered in FBOE research. The odds of homosexual orientation for participants with no older brothers were fixed at 0.02 (this is a baseline estimate that occurs throughout the FBOE literature; e.g., Cantor et al., 2002). Each older brother increased these odds by a factor of $(1 + \theta)$ with $\theta \in (-1,\infty)$. Thus, individuals' simulated odds of homosexual orientation were given by

$$0.02(1 + \theta)^{\#OB}.$$

We investigated the choices of -0.33, 0.33, and 0 as possible values for θ, with 0 representing no effect of older brothers on homosexual orientation at all, -0.33 a decrease in the odds of homosexual orientation per each older brother, and 0.33 a true increase in the odds of homosexual orientation per each older brother. A value of θ = 0.33 corresponds to the 33% increase in the odds of homosexual orientation per older brother reported throughout the literature (Blanchard, 2001; Blanchard et al., 1996); a value of $\theta = 0$ corresponds to a scenario in which the theoretical estimand is 0 and thus, there is no distinct association between the number of older brothers and sexual orientation and a value of θ = -0.33 corresponds to a scenario in which the number of older brothers decreases the odds of homosexual orientation.

The Poisson distribution was parametrised by a vector **λ** containing four elements: The mean numbers of older brothers, older sisters, younger brothers, and younger sisters, respectively (i.e., the *rates* at which these events occur). This vector of means (or rates) was determined by multiplying the mean number of all siblings, μ, by the vector

$$[.515 \times \pi \quad (1 - .515) \times \pi \quad .515 \times (1 - \pi) \quad (1 - .515) \times (1 - \pi)]^T,$$

where .515 again represented the population estimate for the probability of male birth (Grech & Mamo, 2020), and π denoted the proportion of older siblings in a given sample.

For instance, for an average number of siblings of $\mu = 3$ and equal probability that each sibling is an older or a younger sibling (i.e., π = .5), **λ** would be given by

$$\begin{aligned} \boldsymbol{\lambda} &= 3 \times [.515 \times .5 \quad (1 - .515) \times .5 \quad .515 \times (1 - .5) \quad (1 - .515) \times (1 - .5)]^T \\ &= [0.77 \quad 0.73 \quad 0.77 \quad 0.73]^T. \end{aligned}$$

By plugging in different values for π, the simulation could generate an older sibling effect independently of the specific older brother effect θ. This way, we could flexibly adjust the magnitude of θ, while being able to distinguish it from a more general older sibling effect. There are a multitude of alternative ways for implementing different older brothers and older sibling effects in a simulation: This is just one of them. For the homosexual sample, the study employed three different values for π, namely $\pi = .5$, $\pi = .6$, and $\pi = .7$, while for the heterosexual sample π was fixed at .5. That is, we simulated differences in the number (or proportion) of older siblings by increasing π in the homosexual group, assuming that the proportion of older siblings is equal to the proportion of younger siblings among homosexual individuals. We considered only scenarios in which the proportion of older siblings is greater in the homosexual versus the heterosexual sample, because the greater number (or proportion) of older siblings (as opposed to just a greater number of older brothers) represents the alternative model consistent with much of the FBOE literature. Other scenarios, wherein there are fewer older siblings in homosexual as opposed to heterosexual men, may be interesting, but would correspond to a model of homosexual men having fewer older siblings than heterosexual men. This model does not compete with the FBOE model and therefore is irrelevant.

The median of the mean numbers of all siblings for homosexual participants across the 45 samples in Blanchard (2018a, 2018b), and Blanchard et al. (2021) was 2.45. In the *equal* condition of the simulation study, this value served as the mean number of all siblings (i.e., $\mu = 2.45$) for both the homosexual and heterosexual group. In the *unequal* condition, the mean number of siblings for each group were taken from the *Mismatch 2* sample in Blanchard (2014), where the mean number of siblings in the homosexual group was $\mu = 2.19$, and the mean number of siblings in the heterosexual group was $\mu = 3.31$. Blanchard used the *Mismatch 2* sample to demonstrate the inability of tests for mean differences and logistic regression to detect an older brother effect and to promote the use of the *MROB* and *MPOB*.

As mentioned in the introduction in the main text, it is claimed that due to a positive correlation between the number of older brothers and older sisters, homosexual men should also have more older sisters than heterosexual men (Blanchard, 2014; Blanchard & Klassen, 1997; Jones & Blanchard, 1998). However, to our knowledge, no estimate of the magnitude, or an explanation of the precise nature, of the correlation, that would be required to bring about a difference in the number of older sisters, has been put forth. We nevertheless address this purported correlation and incorporated our best guess of such an estimate, by imposing the correlation matrix of the four sibling types found in the male participants of Tran et al. (2019) onto our simulated participants (Table S2; see https://osf.io/3wnhu/ for data). This study consisted of 1,779 men, who provided complete information on the number of each sibling type (see the original publication for full description of the sample). That is, the simulation assumed equal correlation structures in both homosexual and heterosexual participants, as we are unaware of any claims in the FBOE literature that these structures should differ between homosexual and heterosexual men.

Thus, there were 18 possible combinations of θ, μ and π. For each combination, we fitted 10 different models (see Table 3 in the main text).

Table S2. *Correlation matrix used to simulate participants.*

	#OB	#OS	#YB	#YS
#OB	1			
#OS	.158	1		
#YB	-.092	-.071	1	
#YS	-.057	-.066	.063	1

Note. #OB = Number of older brothers, #OS = Number of older sisters, #YB = Number of younger brothers, #YS = Number of younger sisters. Correlations taken from Tran et al. (2019), see https://osf.io/3wnhu/ for the raw data.

S5.2 Further Evaluation and Sample-Size Considerations

The performance of the models was primarily assessed with respect to inferring the state of θ with null-hypothesis significance testing (NHST), as it is the case throughout the FBOE literature. Only Models 1, 6, 7, and 9 in Table 3 in the main text could be interpreted as providing an estimate of θ (by exponentiating the estimates and subtracting 1). It thus makes little sense to report conventional quantities such as bias, (root) mean-squared errors, or coverage probabilities, which are used to assess how well the coefficients of interest of the models in Table 3 perform in estimating θ, as most of the model coefficients of interest in Table 3 are not intended to estimate θ or used in that manner. For instance, it would not be meaningful to determine the relative frequency (i.e., the coverage probability) with which the natural logarithm (ln) of the factor (1+θ) (i.e., the odds ratio for being homosexual per additional older brother) is inside the 95% *CI* for the ln*OBOR* (Model 4), as the ln*OBOR* is an entirely different unknown parameter than ln(1+ θ). Say, ln*OBOR* = ln(1+η), where η is the proportional increase in the odds of observing an older brother in the homosexual versus heterosexual group, whereas θ is the proportional increase in the odds of being homosexual per older brother. It is evident that η and θ are entirely different parameters.

Owing to the finite number of observations (1,000 replications per condition), the observed false-positive rates for the regression coefficients could differ from the underlying real false-positive rate. We defined the margin of error for an acceptable true false-positive rate of .05 as follows: Falsely rejected null-hypotheses can be modelled as Bernoulli random variables with probability of success equal to .05. Thus, the sum of 1,000 Bernoulli random variables (i.e., the number of falsely rejected null-hypotheses) is a binomial random variable. Suppose a test has a false-positive rate of 5%. The probability of observing 67 successes or less in 1000 trials (i.e., a false-positive rate of 6.7%) is .99. Hence, we regarded an observed false-positive rate of greater than 5%, but smaller than 6.7% as compatible with a true false-positive rate of 5%. Sample sizes were determined by carrying out the simulation several times, increasing the sample size on each iteration until at least 80% of the regression coefficients for the predictor #OB in Model 9 returned a $p < .05$ (two-tailed) given θ = 0.33. In other words, we increased the sample size until the coefficient of interest in Model 9 had a minimum approximate power of 80% or more. Conditional on the alternative hypothesis (here: $\theta = 0.33$) being true, a test is equivalent to a Bernoulli random variable with probability of success equal to the test's power. Thus, the probability

of observing 829 successes or less in 1,000 trials (i.e., a success rate of 82.9%) is .99. It should then be safe to conclude that for an observed rate of correctly rejected null hypotheses of 829 across 1,000 simulation replications, the hypothesis tests had a power of at least 80%. A sample size of 700 participants per group seemed to be more than sufficient for this purpose (see results below). The rationale for tuning the sample size such that Model 9 had adequate power was that this model not only adjusted for the number of older siblings but also provided a direct estimate for θ (i.e., the older brother effect in our simulation study) and was thus the most easily interpretable of the models.

S5.3 Models

Model 1 in Table 3 in the main text predicted the logit of the probability of being homosexual, using the raw number of older brothers and older sisters as predictors. Blanchard (2014) warned against using this model, stating that differences in the total number of siblings between homosexual and heterosexual participants (i.e., heterosexual participants having more siblings in general) may lead to the non-detection of a genuine association between the number of older brothers and sexual orientation as conveyed by the regression coefficient of #OB.

Models 2 and 3 used the *MROB* and *MROS* (Model 2) or the *MPOB* and *MPOS* (Model 3), respectively, instead of the raw numbers of older brothers and older sisters as predictors in a logistic regression model, as recommended by Blanchard (2014) and Blanchard et al. (2020).

The regression coefficients for the predictor Hom_i in Models 4 and 5 correspond to the natural logarithms (ln) of the *OBOR* (Model 4) and the *OSOR* (Model 5) when regarded as generalized linear models at the level of the reported siblings. Specifically, Model 4 predicted the logit probability of the jth sibling reported by the ith participant being an older brother (for Model 5: older sister), using only the homosexual orientation of the ith participant as a predictor (abbreviated as "Hom"), with a value of "0" meaning no homosexual orientation and a value of "1" representing homosexual orientation. In Model 5, $\exp(\beta_{\text{Hom}})$ was not intended to capture the older-brother effect (θ) itself, but to assess a positive, but weaker, *OSOR* (as compared to the *OBOR*), which should be observable in the presence of an isolated effect of older brothers on sexual orientation, presumably due to the positive correlation between older brothers and older sisters (Blanchard, 2018c; Blanchard et al., 2021).

Models 6 and 7 both included the raw number of older brothers as one predictor, but differed with respect to the second predictor, i.e., the variable whose confounding effect should be adjusted for. Model 6 adjusted for the number of all siblings, #All, and Model 7 adjusted for the number of siblings who are not older brothers (i.e., "other siblings," #Other). These are the models implied by the claimed necessity of having to adjust for #All and #Other (as opposed to the *MPOB* and the *MROB*), respectively.

Models 8 through 10 adjusted for the *number of older siblings*, #Older (Frisch & Hviid, 2006; Gelman & Stern, 2006; Zietsch, 2018). Model 8 corresponds to the one suggested by Gelman and Stern (2006) and included the difference between the number of older brothers and older sisters as the predictor of interest, while adjusting for #Older. The regression

coefficient of interest, $\beta_{\#OB-\#OS}$, quantifies the particular association between older brothers and homosexual orientation. Model 9 contains instead the number of older brothers as the predictor of interest.

Finally, the regression coefficient for Hom_i in Model 10 is equivalent to the natural logarithm (ln) of the odds ratio of older brothers to older sisters introduced in Equation 6, with the sampling frame being restricted to older siblings only. Model 10 predicted the logit of the probability of the jth older sibling reported by the ith participant being an older brother using the homosexual orientation (coded as 0 and 1) of the ith participant.

Thus, just like Models 8 and 9, Model 10 adjusts for the number of older siblings, but is defined on the level of the reported siblings, rather than the level of participants.

S5.4 Summary Plots of Models Fitted to Simulated Data

The full of the seven logistic regression models predicting the probability of homosexual orientation from the simulation study are displayed as error plots in Figure S3. These plots are different from Figures 1 and 2 in the main text, in that they also convey summaries of the regression coefficients of the predictors that the models in Table 3 (main text) were intended to control for. As noted in the main text (Footnote 5), the regression coefficients for the *MROB* and *MPOB* were consistently greater in magnitude than the corresponding regression coefficients for the *MROS* and *MPOS* when an older brother effect was present. This suggests that interpreting the two conjointly (for instance, by considering their difference) may yield decisions with a false-positive rate of 5%.

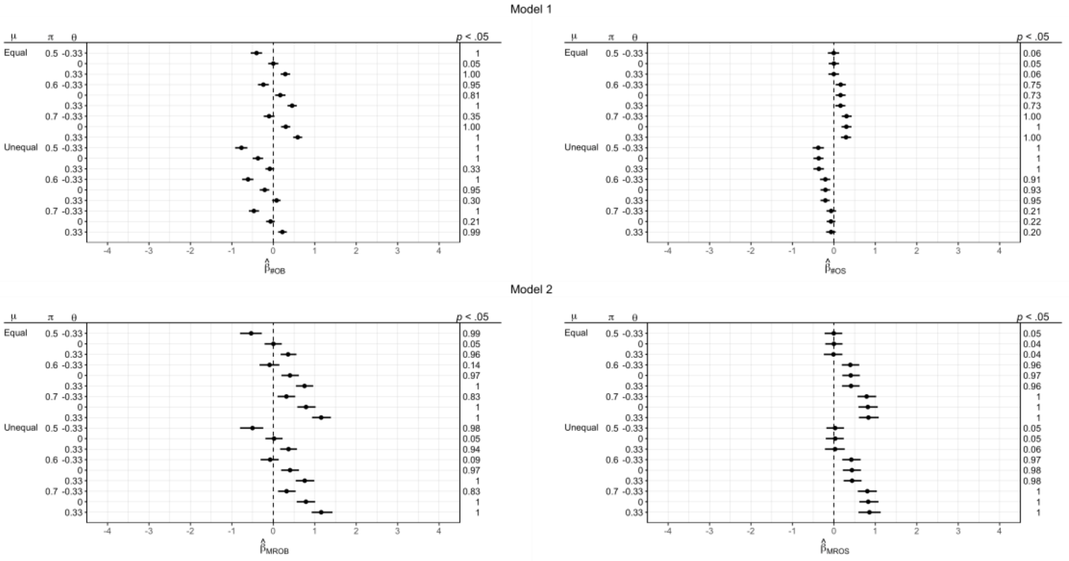

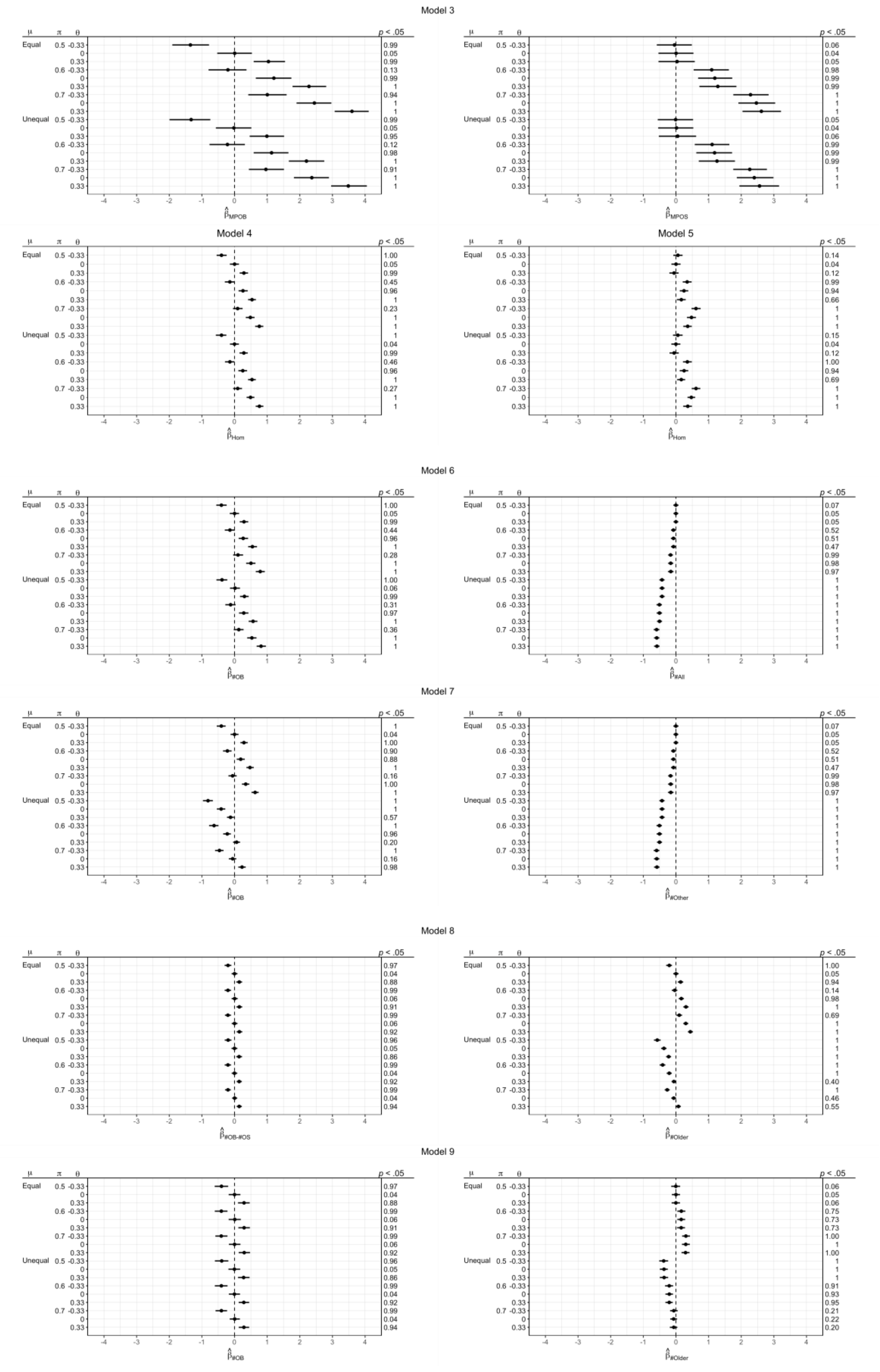
Model 3
Model 4
Model 5
Model 6
Model 7
Model 8
Model 9
μ π θ
p < .05
Equal
Unequal

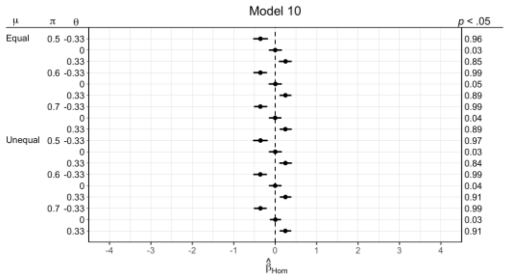

Figure S3. The error plots show the average log odds ratios (ln*OR*) over 1,000 simulations of the seven logistic regression models predicting the probability of homosexual orientation for each combination of μ, π and θ. The column μ indicates whether the mean number of all siblings was equal at μ = 2.25 for both the homo- and heterosexual participants or whether this mean was unequal, i.e., the mean number of all siblings was greater in heterosexual group (μ = 3.31) as opposed to the homosexual group (μ = 2.19). The column π denotes the proportion of older siblings in the homosexual group. For the heterosexual group, π was fixed at 0.5. The column θ denotes the presence of a positive (θ = 0.33), a negative (θ = -0.33) or the absence (θ = 0) of an effect of older brothers on the odds of homosexual orientation. The endpoints of the error bars correspond to the .025 and .975 quantiles of the distribution of simulated coefficients. The column "% p < .05" indicates the percentage of simulation replications that yielded a two-tailed *p*-value of less than .05.

S6 Meta-Analyses in Part III

S6.1 Meta-Analyses of Specific Subsets using the Difference in Proportion

The following provides the *R* code for conducting the meta-analyses of the five specific study sets, using the difference in proportion (as opposed to the ln*OR* in the main text) as an effect size. To this end, it is first needed to compute the proportion of older brothers among the set of older siblings for the homosexual and heterosexual groups in each sample:

```
# Load packages
library(metafor) #Used for fitting meta-analysis

# Load male and female data and compute proporitons of older brothers
# among older siblings and variances
es_male <- read_csv("../Data/maleData.csv") %>%
  mutate(
    prop_ob_hom = obHom/(obHom+osHom),
    prop_ob_het = obHet/(obHet+osHet)
  ) %>%
  mutate(
    prop_ob_hom_vi = 1/(obHom+osHom)*prop_ob_hom*(1-prop_ob_hom),
    prop_ob_het_vi = 1/(obHet+osHet)*prop_ob_het*(1-prop_ob_het)
  ) %>%
  mutate(
    prop_diff = prop_ob_hom-prop_ob_het,
```

```
    prop_diff_vi = prop_ob_hom_vi+prop_ob_het_vi
  )


es_female <- read_csv("../Data/femaleData.csv") %>%
  mutate(
    prop_ob_hom = obHom/(obHom+osHom),
    prop_ob_het = obHet/(obHet+osHet)
  ) %>%
  mutate(
    prop_ob_hom_vi = 1/(obHom+osHom)*prop_ob_hom*(1-prop_ob_hom),
    prop_ob_het_vi = 1/(obHet+osHet)*prop_ob_het*(1-prop_ob_het)
  ) %>%
  mutate(
    prop_diff = prop_ob_hom-prop_ob_het,
    prop_diff_vi = prop_ob_hom_vi+prop_ob_het_vi
  )
```

The random effects estimate for the mean proportion of older brothers among the older siblings reported by heterosexual group was .505, 95% *CI* = [.495, .516]. To reproduce this result, call:

```
rma(yi = prop_ob_het, vi = prop_ob_het_vi, data = es_male, slab = study)


Random-Effects Model (k = 64; tau^2 estimator: REML)

tau^2 (estimated amount of total heterogeneity): 0.0008 (SE = 0.0003)
tau (square root of estimated tau^2 value):      0.0282
I^2 (total heterogeneity / total variability):   89.99%
H^2 (total variability / sampling variability):  9.99

Test for Heterogeneity:
Q(df = 63) = 1112.6991, p-val < .0001

Model Results:

estimate      se     zval    pval   ci.lb   ci.ub
  0.5054  0.0051  98.8366  <.0001  0.4954  0.5155  ***


---
Signif. codes:  0 '***' 0.001 '**' 0.01 '*' 0.05 '.' 0.1 ' ' 1
```

The fixed-effect estimate, however, was quite different from this value (and also from the theoretically expected value of .515)

```
(
  fe_prop <-
    rma(
      yi = prop_ob_het,
      vi = prop_ob_het_vi,
```

```
      data = es_male,
      method = "FE",
      slab = study
    )
)


Fixed-Effects Model (k = 64)

I^2 (total heterogeneity / total variability):   94.34%
H^2 (total variability / sampling variability):  17.66

Test for Heterogeneity:
Q(df = 63) = 1112.6991, p-val < .0001

Model Results:

estimate      se      zval     pval    ci.lb    ci.ub
  0.5441  0.0008  670.8997  <.0001  0.5426  0.5457  ***

---
Signif. codes:  0 '***' 0.001 '**' 0.01 '*' 0.05 '.' 0.1 ' ' 1
```

This deviation from .515 of almost 3 percentage points seemed to have been largely due to the sample by Frisch and Hviid (2006), as removing this sample from the analysis resulted in fixed-effects estimates much closer to .515. To see how the meta-analytic estimates change upon the exclusion of a sample, we invoke the `leave1out()` function, which fits separate meta-analyses for each subset of samples, leaving out one sample at a time. The resulting table of meta-analytic summaries (each row corresponds to one of the leave-one-out meta-analyses) is sorted in ascending order of the meta-analytic summary estimate.

```
arrange(as.data.frame(leave1out(fe_prop))[, c("estimate", "ci.lb", "ci.ub")],
estimate) %>%
  round(3) %>%
  head()

                                estimate ci.lb ci.ub
Frisch and Hviid (2006)            0.506 0.503 0.509
Blanchard et al. (1996) Study 2    0.544 0.543 0.546
VanderLaan et al. (2016)           0.544 0.543 0.546
Blanchard et al. (1996) Study 1    0.544 0.543 0.546
Blanchard and Bogaert (1998) OP    0.544 0.543 0.546
Blanchard and Zucker (1994)        0.544 0.543 0.546
```

With reference to the theoretical value of .515, the proportions of older brothers among older sisters reported by Frisch and Hviid (2006) were elevated in both, the homo- and heterosexual men at .55 and .56, respectively (i.e., almost 5 percentage points from the expected proportion). To see this, simply call

```
es_male$prop_ob_het[es_male$study=="Frisch and Hviid (2006)"]
```

```
[1] 0.56037

es_male$prop_ob_hom[es_male$study=="Frisch and Hviid (2006)"]

[1] 0.5521327
```

For the heterosexual female samples, the fixed- and random-effects estimates for the mean proportion of older brothers among older siblings are obtained by calling:

```
# Random-Effects
rma(
  yi = prop_ob_het,
  vi = prop_ob_het_vi,
  data = es_female,
  slab = study
)


Random-Effects Model (k = 17; tau^2 estimator: REML)

tau^2 (estimated amount of total heterogeneity): 0.0017 (SE = 0.0008)
tau (square root of estimated tau^2 value):      0.0409
I^2 (total heterogeneity / total variability):   98.18%
H^2 (total variability / sampling variability):  55.06

Test for Heterogeneity:
Q(df = 16) = 308.5711, p-val < .0001

Model Results:

estimate      se     zval    pval   ci.lb   ci.ub
  0.5005  0.0123  40.7806  <.0001  0.4765  0.5246  ***

---
Signif. codes:  0 '***' 0.001 '**' 0.01 '*' 0.05 '.' 0.1 ' ' 1

# Fixed-Effects
(
  fe_female <- rma(
    yi = prop_ob_het,
    vi = prop_ob_het_vi,
    data = es_female,
    slab = study,
    method = "FE"
  )
)


Fixed-Effects Model (k = 17)

I^2 (total heterogeneity / total variability):   94.81%
```

```
H^2 (total variability / sampling variability):  19.29

Test for Heterogeneity:
Q(df = 16) = 308.5711, p-val < .0001

Model Results:

estimate      se      zval    pval   ci.lb   ci.ub
  0.5465  0.0008  683.0655  <.0001  0.5449  0.5480  ***


---
Signif. codes:  0 '***' 0.001 '**' 0.01 '*' 0.05 '.' 0.1 ' ' 1
```

And again, the Frisch and Hviid (2006) sample seems to be responsible for the discrepancy between the fixed- and random-effects estimates:

```
arrange(as.data.frame(leave1out(fe_prop))[, c("estimate", "ci.lb", "ci.ub")],
estimate) %>%
  round(3) %>%
  head()

                                estimate ci.lb ci.ub
Frisch and Hviid (2006)            0.506 0.503 0.509
Blanchard et al. (1996) Study 2    0.544 0.543 0.546
VanderLaan et al. (2016)           0.544 0.543 0.546
Blanchard et al. (1996) Study 1    0.544 0.543 0.546
Blanchard and Bogaert (1998) OP    0.544 0.543 0.546
Blanchard and Zucker (1994)        0.544 0.543 0.546
```

Next, we compute the difference in proportions and the corresponding variance (or standard error) for each sample under the assumption that within-study dependence of these proportions is negligible.

```
es_male <- es_male %>%
  mutate(
    prop_diff = prop_ob_hom-prop_ob_het,
    prop_diff_vi = prop_ob_hom_vi+prop_ob_het_vi
  )

es_female <- es_female %>%
  mutate(
    prop_diff = prop_ob_hom-prop_ob_het,
    prop_diff_vi = prop_ob_hom_vi+prop_ob_het_vi
  )
```

The meta-analyses for each of the five specific study sets are obtained by calling

```
# Create list of subsets
exemp_set <- list(
  blanchard_2018 = es_male[es_male$inD==1, ],
  blanchard_2018b = es_male[es_male$inE==1, ],
```

```
  blanchard_2020 =es_male[es_male$inG==1, ],
  full_set = es_male,
  female = es_female
)

# Vector of methods "FE" = fixed effects, "REML" = restr. max. likel.
method <- c("FE", "REML")

# Results are stored in list "res"
res <- list()
for (i in 1:length(method)) {
  if (method[i] == "FE") {
    res[[i]] <- lapply(exemp_set, function(y) {
      rma(
        yi = prop_diff,
        vi = prop_diff_vi,
        slab = study,
        method = method[i],
        data = y
      )
    })
  } else {
    res[[i]] <- lapply(exemp_set, function(y) {
      rma.mv(
        yi = prop_diff,
        V = prop_diff_vi,
        slab = study,
        random = ~ 1 | as.factor(study),
        data = y
      )
    })
  }
}

# Meta-analytic summaries will be stored in list "res_tbl"
res_tbl <- list()

for(i in 1:length(method)){
  if(method[i] == "FE"){
    res_tbl[[i]] <- lapply(res[[i]], function(x) {
      data.frame(
        rbind(
          mean = as.matrix(predict(x))[,c(1,3,4)]
        )
      ) %>% set_names(c("estimate", "2.5%", "97.5%")) %>%
        round(3)
    })
  } else {
    res_tbl[[i]] <- lapply(res[[i]], function(x) {
      data.frame(
```

```
        rbind(
          mean = as.matrix(predict(x))[,c(1,3,4)],
          tau = confint(x)[[1]][2,],
          predicted = as.matrix(predict(x))[,c(1,5,6)]
        )) %>% set_names(c("estimate", "2.5%", "97.5%")) %>%
        round(3)
    })
  }
}
```

The estimates for the fixed-effects models are:

```
res_tbl[[1]]

$blanchard_2018
     estimate  2.5% 97.5%
mean    0.036 0.022  0.05

$blanchard_2018b
     estimate   2.5% 97.5%
mean    0.008 -0.016 0.031

$blanchard_2020
     estimate 2.5% 97.5%
mean    0.025 0.01  0.04

$full_set
     estimate  2.5% 97.5%
mean    0.017 0.009 0.024

$female
     estimate  2.5% 97.5%
mean    0.017 0.005 0.029
```

Similarly, the estimates for the random-effects models are:

```
res_tbl[[2]]

$blanchard_2018
          estimate   2.5% 97.5%
mean         0.043  0.022 0.065
tau          0.038  0.000 0.068
predicted    0.043 -0.034 0.121

$blanchard_2018b
          estimate   2.5% 97.5%
mean         0.032 -0.014 0.078
tau          0.037  0.000 0.114
predicted    0.032 -0.055 0.118

$blanchard_2020
```

```
            estimate 2.5% 97.5%
mean           0.025 0.01 0.040
tau            0.000 0.00 0.031
predicted      0.025 0.01 0.040

$full_set
            estimate   2.5% 97.5%
mean           0.033  0.018 0.048
tau            0.036  0.018 0.057
predicted      0.033 -0.040 0.106

$female
            estimate   2.5% 97.5%
mean           0.019 -0.031 0.069
tau            0.073  0.026 0.138
predicted      0.019 -0.134 0.171
```

Finally, fixed-effects, random-effects, and three-level models, comparing the effect in men and women, are fitted:

```
# combine the two samples
es_full <-
  rbind(es_male[, c("prop_diff", "prop_diff_vi", "ref")],
        es_female[, c("prop_diff", "prop_diff_vi", "ref")])

# Add 'sex' and ID as a factor
es_full$sex <- c(rep(1, nrow(es_male)), rep(0,nrow(es_female)))
es_full$id <- 1:nrow(es_full)

# Fixed-Effects Model
rma(
  yi = prop_diff,
  vi = prop_diff_vi,
  data = es_full,
  mods = ~ as.factor(sex),
  method = "FE"
)


Fixed-Effects with Moderators Model (k = 81)

I^2 (residual heterogeneity / unaccounted variability): 46.76%
H^2 (unaccounted variability / sampling variability):   1.88
R^2 (amount of heterogeneity accounted for):            0.00%

Test for Residual Heterogeneity:
QE(df = 79) = 148.3965, p-val < .0001

Test of Moderators (coefficient 2):
QM(df = 1) = 0.0080, p-val = 0.9286
```

```
Model Results:

                  estimate      se     zval    pval    ci.lb   ci.ub
intrcpt             0.0171  0.0060   2.8484  0.0044   0.0053  0.0289  **
as.factor(sex)1    -0.0006  0.0072  -0.0895  0.9286  -0.0147  0.0134

---
Signif. codes:  0 '***' 0.001 '**' 0.01 '*' 0.05 '.' 0.1 ' ' 1

# Random-Effects Model
rma.mv(
  yi = prop_diff,
  V = prop_diff_vi,
  data = es_full,
  random = ~1|id,
  mods = ~as.factor(sex)
)


Multivariate Meta-Analysis Model (k = 81; method: REML)

Variance Components:

            estim    sqrt  nlvls  fixed  factor
sigma^2    0.0018  0.0419     81     no      id

Test for Residual Heterogeneity:
QE(df = 79) = 148.3965, p-val < .0001

Test of Moderators (coefficient 2):
QM(df = 1) = 0.8080, p-val = 0.3687

Model Results:

                  estimate      se    zval    pval    ci.lb   ci.ub
intrcpt             0.0160  0.0182  0.8784  0.3797  -0.0197  0.0518
as.factor(sex)1     0.0180  0.0200  0.8989  0.3687  -0.0213  0.0573

---
Signif. codes:  0 '***' 0.001 '**' 0.01 '*' 0.05 '.' 0.1 ' ' 1

# 3-level model
rma.mv(
  yi = prop_diff,
  V = prop_diff_vi,
  data = es_full,
  random = list(~1|id, ~1|as.factor(ref)),
  mods = ~as.factor(sex)
)
```

```
Multivariate Meta-Analysis Model (k = 81; method: REML)

Variance Components:

             estim    sqrt  nlvls  fixed          factor
sigma^2.1  0.0009  0.0293     81     no              id
sigma^2.2  0.0011  0.0326     53     no  as.factor(ref)

Test for Residual Heterogeneity:
QE(df = 79) = 148.3965, p-val < .0001

Test of Moderators (coefficient 2):
QM(df = 1) = 0.7577, p-val = 0.3840

Model Results:

                 estimate      se    zval    pval    ci.lb   ci.ub
intrcpt            0.0173  0.0177  0.9749  0.3296  -0.0174  0.0520
as.factor(sex)1    0.0159  0.0183  0.8705  0.3840  -0.0199  0.0518

---
Signif. codes:  0 '***' 0.001 '**' 0.01 '*' 0.05 '.' 0.1 ' ' 1
```

S6.2 Results of the Fixed-Effect Model Analyses

Table S3 presents the fixed-effect summary estimates of the four sets of male samples and the full set of female samples.

Table S3. *Summary of results of fixed-effect meta-analyses using either lnOR or lnOBOR as an effect size.*

Set	Unit: ln*OR*	Unit: ln*OBOR*
Men included in Blanchard (2018a)	0.14 [0.08, 0.20]	0.32 [0.28, 0.37]
Men included in Blanchard (2018b)	0.03 [-0.07, 0.12]	0.20 [0.12, 0.27]
Men included in Blanchard et al. (2021)	0.10 [0.04, 0.16]	0.25 [0.20, 0.30]
Men Full Set	0.06 [0.03, 0.09]	0.28 [0.25, 0.32]
Women full set	0.07 [0.02, 0.12]	0.15 [0.07, 0.23]

Note. Numbers are estimated fixed-effect means with 95% confidence intervals.

The difference between male and female samples amounted to -0.004, 95% *CI* = [-0.061, 0.053].

The re-analysis of Blanchard (2018a) using the ln*OR* returned fixed-effect summary estimates of 0.09, 95% *CI* = [0.03, 0.16], for the "non-feminine/cisgender" subgroup of samples and 0.29, 95% *CI* = [0.17, 0.40], for the "feminine" group. Across all 47 samples which were not denoted "feminine" by Blanchard (2018a), we obtained fixed-effect estimates of ln*OR* = 0.05, 95% *CI* = [0.01, 0.08].

Regressing the observed effect sizes on their standard errors, the regression coefficient for the standard error was 0.70, 95% *CI* = [0.35,1.05] in the fixed-effect model. This indicates that (when rescaling these coefficients by dividing them by 10), on average, two effect sizes differing by 0.1 standard errors are expected to differ by 0.07 ln*OR*s. Sample size contributes less to study weight in the random-effects model than in the fixed-effect model. Hence, small-study effects (if they go into the direction of the desired effect, as was the case in the present data) may explain the larger summary estimates in the random-effects models (Table 7 in the main text), compared to those of the fixed-effect models in Table S4.

In the specification curve and multiverse analyses, when including GDY as a predictor, the fixed-effect summary estimates for the 52 non-GDY and the 12 GDY samples were ln*OR* = 0.05, 95% CI = [0.02, 0.08], and ln*OR* = 0.30, 95% CI = [0.16, 0.44]. Including the Which factor *in Previous MA* as a moderator in the meta-analysis consisting of all 64 samples, the fixed-effect summary estimates were ln*OR* = 0.02, 95% *CI* = [-0.02,0.07], and ln*OR* = 0.11, 95% *CI* = [0.06,0.16] (difference of 0.09, 95% *CI* = [0.03,0.15], in favour of studies included in previous meta-analyses).

Figure S4 presents a rainforest plot of the fixed-effect meta-analysis of all male samples ln*OR* effect sizes.

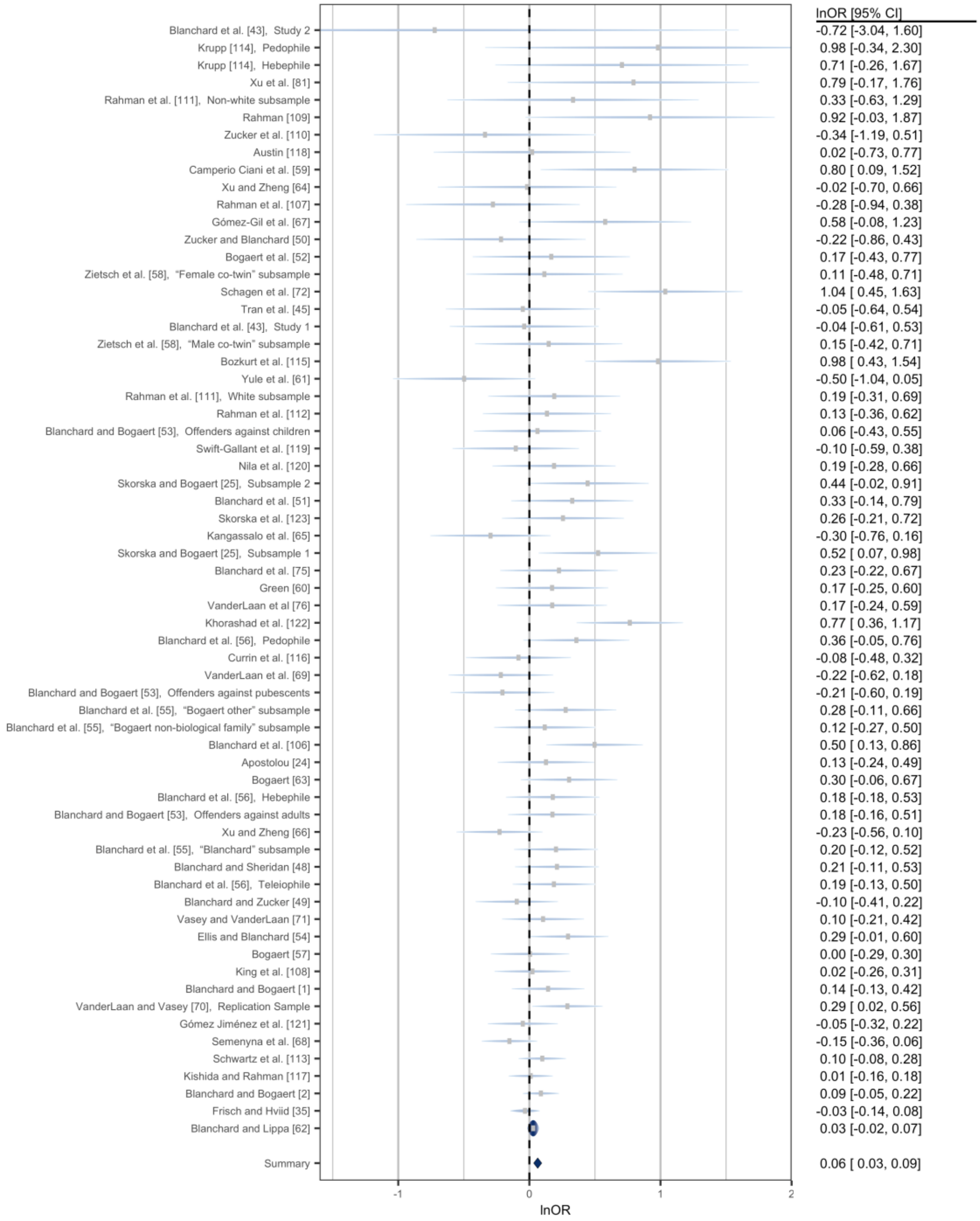

Figure S4. Rainforest plot of the fixed-effect meta-analysis of all male sample lnOR effect sizes. In this plot, the sample weights are both encoded in the degree of saturation and the thickness of the shaded regions, with heavier effect sizes being more saturated and thicker. The widths of the shaded regions correspond to 95% confidence intervals (*CIs*); the point estimates are represented by the ticks inside these intervals. The teardrop-shape encodes the relative likelihood function of the mean of a normal distribution conditional on the standard deviation being equal to the observed standard error (i.e., the ln*ORs* are assumed to be distributed normally) and its horizontal mirror image over the range of the 95% *CI*. The teardrop shape encodes the likelihood of true values over the

range of the 95% CI, given the observed estimate and assuming the lnORs to be normally distributed with known variance.

S7 Distribution of *S* Values Under the Null Model

Technically, if a test statistic is continuous (as opposed to discrete) and the null (or test) hypothesis refers to a single parameter value (e.g., H_0: $\beta = \beta_0$), the associated p value is distributed uniformly over the unit interval upon repeated sampling if the null model is true.

Let U be distributed uniformly over the unit interval, i.e., $U \sim \text{Unif}(0,1)$, let $S = \log_2(1/U)$, and denote by F_S and F_U the cumulative distribution functions (cdf) of S and U, respectively. Then

$$
\begin{aligned}
F_S(s) \quad &= P(S \leq s) \\
&= P(\log_2(1/U) \leq s) \\
&= P(1/U \leq 2^s) \\
&= P(U > 2^{-s}) \\
&= 1 - F_U(2^{-s}) \\
&= 1 - 2^{-s} \\
&= 1 - \exp\{-\ln(2)s\},
\end{aligned}
$$

which describes the cdf of an exponential distribution with rate parameter equal to $\ln(2)$; $\text{Exponential}\{\lambda = \ln(2)\}$. Thus, under the null model and given a continuous test statistic, S values follow an exponential distribution.

S8 Parametric Bootstrapping Plots

A modified approach to the parametric bootstrapping procedure described in Voracek et al. (2019) was carried out to also explore its sensitivity to different degrees of effect size heterogeneity. For each of the 64 male samples, a random value from a normal distribution with mean equal to zero and variance equal to $\sigma_j^2 + \tau^2$ was drawn. Three τ values were used: (1) $\tau = 0$, corresponding to a fixed-effect model; (2) $\tau = 0.13$, corresponding to the REML-estimate observed across all 64 male samples; (3) $\tau = 0.21$, corresponding to the upper bound of the 95% CI for the REML-estimate. We computed the 1,638 meta-analytic specifications for each set of simulated effect sizes, and repeated this procedure 1,000 times. The resulting 0.025 and 0.975 quantiles of the estimated meta-analytic means for each of the 1,638 specification were sorted in ascending order. Plotting each specification's quantiles and connecting them with a line resulted in a 95% parametric bootstrap confidence region for specification curves under the null model. Figure S5 displays the results of these simulations for both male and female samples.

The observed specification curve (red line) for male samples (Figure S5, top row) is mostly located outside the bootstrapped confidence bands created by the simulated data under the three conditions of effect size heterogeneity considered here. This suggests a mismatch

between the data and the respective models which entail an effect of lnOR = 0. This is in line with the results obtained thus far – conditional on all assumptions about the data generating process being met (most importantly, the assumption that the observed effect sizes are a random selection of all effect sizes; e.g., Rafi & Greenland, 2020) the effect does not appear to be zero (or in the immediate vicinity of zero).

In comparing the female specification curve to its respective confidence bands, it is easy to see that most of the meta-analytic effects are well inside the confidence bands for the null model which assumes an lnOR of equal to 0. Thus, the meta-analytic summary effects of the specifications of female samples do not appear unusual given the null model.

Note that it would be a mistake to conclude that (a) “the effect is present in male samples but not in female samples” or (b) “the effect is greater in male as opposed to female samples”. As far as (a) is concerned, the confidence bands could theoretically be made arbitrarily narrow if enough female samples were available (no effect is exactly zero). As far as (b) is concerned: Judgments about the size of a difference between two effects actually require that this difference be assessed - otherwise one would fall victim to the “the-difference-between-significant-and-not-significant-is-not-itself-statistically-significant”-fallacy (Gelman & Stern, 2006; the inferential specification curves are in fact equivalent to hypothesis tests for each observed specification summary effect).

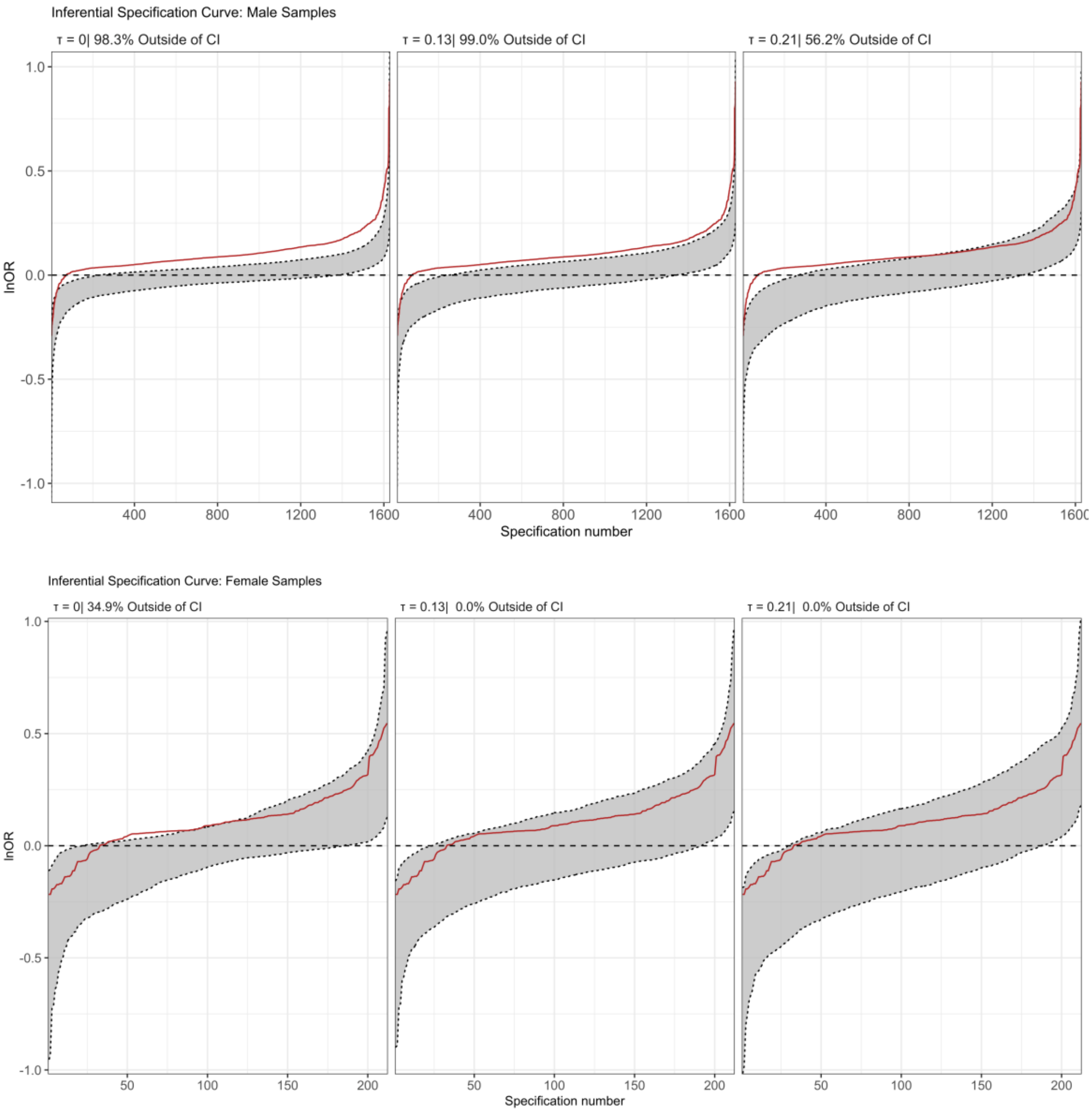

Figure S5. Inferential specification curve plots for the male (top) and female (bottom) samples and three values of τ;. The plots show the observed specification curves from Figures 5 and 6 (red lines) in the main text for male and female samples. The lower and upper bounds of the grey area correspond to the .025 and .975 quantiles of 1,000 simulations of each specification obtained by drawing effect size estimates from a normal distribution with mean equal to 0, heterogeneity equal to τ; (= 0, 0.13, 0.19), and standard deviation equal to the observed standard error. Observed estimates outside this area are taken as evidence against a true effect of $\ln OR = 0$.

S9 Specification Curve Analysis over the Difference of Male and Female Samples

Here we present the results of specification-curve multiverse meta-analyses obtained by comparing the estimated meta-analytic means for each of the 1,638 specifications of male samples to the fixed- and random-effects mean for all 17 female samples. To this end, we added all 17 female samples to each of the 814 unique subsets of male samples and computed both fixed- and random-effects meta-analyses using sex (male versus female) as a predictor. The resulting 1,628 regression coefficients, (i.e., the difference in the mean effect between male and female samples for each specification), the 95% *CI*s and the *p* values were extracted. Figure S6 shows the corresponding specification-curve (see Part III in the main text for description of specification-curve plot).

The estimated mean differences between the male subsets and all 17 female samples ranged from ln*OR* = -0.36 to ln*OR* = 0.86, with an inter-quartile range of -0.01 to 0.08. 69% of these differences were greater than 0. Of these, 4.3% were accompanied by 95% *CI*s which did not include 0 (i.e., had a two-tailed *p* value of less than .05). Overall, 3.3% of specifications returned $p < .05$.

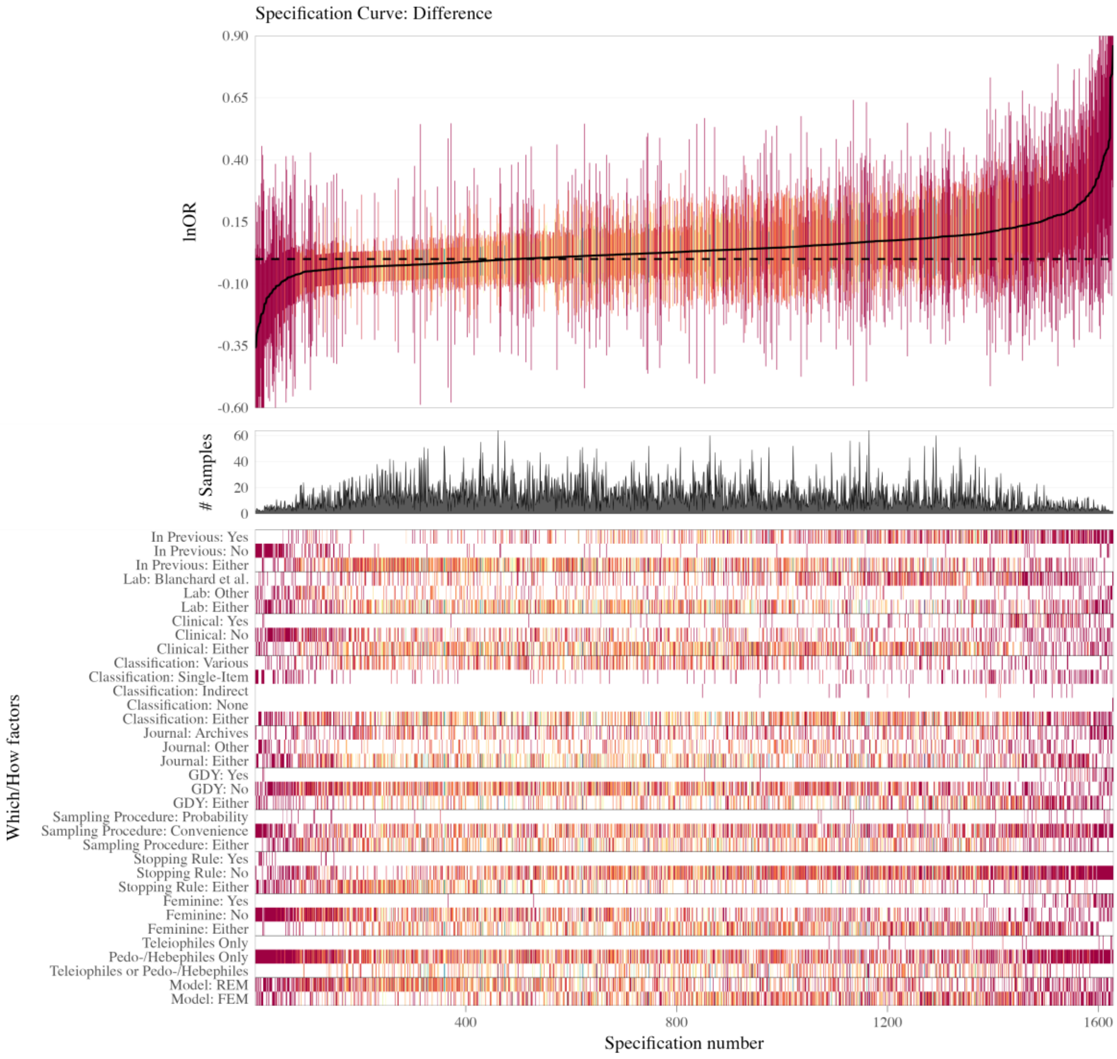

Figure S6. Specification-curve for the difference between male and female samples.

Figure S7 provides the inferential specification-curve plots, Figure S8 the kernel-density estimates of the *S* values.

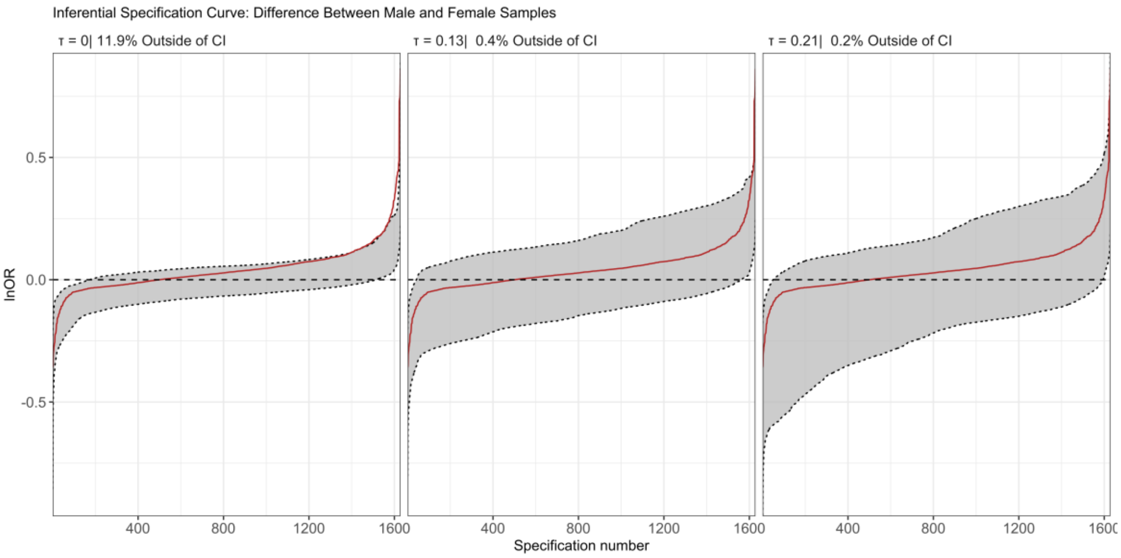

Figure S7. Inferential specification-curve plots for the difference between male and female samples and three values of τ.

The kernel density estimate of the of S-values shown in Figure S8 appears to be reasonably well approximatedby an exponential distribution with rate parameter $\lambda = \ln(2)$, as judged by the visual comparison of the observed S values to the 1,638 random draws from an Exponential$\{\ln(2)\}$ distribution as well as the probability density function.

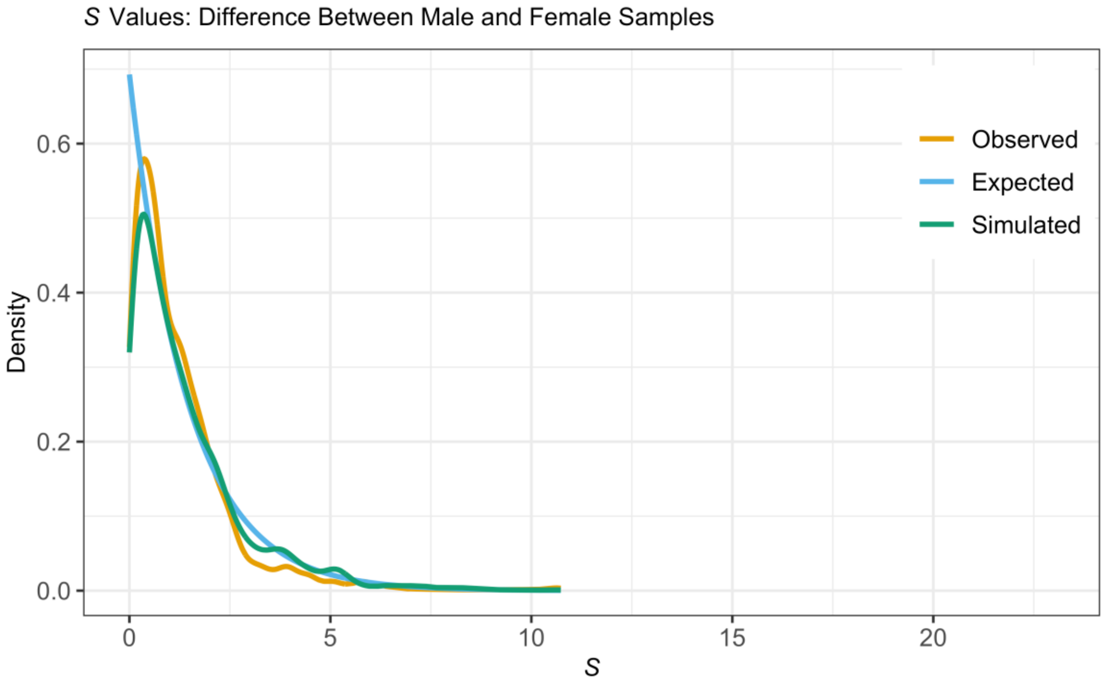

Figure S8. Kernel density estimate of the S values for the difference between male and female samples.

References

Blanchard, R. (2001). Fraternal birth order and the maternal immune hypothesis of male homosexuality. *Hormones and Behavior*, *40*(2), 105–114. https://doi.org/10.1006/hbeh.2001.1681

Blanchard, R. (2004). Quantitative and theoretical analyses of the relation between older brothers and homosexuality in men. *Journal of Theoretical Biology*, *230*(2), 173–187. https://doi.org/10.1016/j.jtbi.2004.04.021

Blanchard, R. (2008). Review and theory of handedness, birth order, and homosexuality in men. *Laterality Asymmetries of Body, Brain and Cognition*, *13*(1), 51–70. https://doi.org/10.1080/13576500701710432

Blanchard, R. (2014). Detecting and correcting for family size differences in the study of sexual orientation and fraternal birth order. *Archives of Sexual Behavior*, *43*(5), 845–852. https://doi.org/10.1007/s10508-013-0245-3

Blanchard, R. (2018a). Fraternal birth order, family size, and male homosexuality: Meta-Analysis of studies spanning 25 years. *Archives of Sexual Behavior*, *47*(1), 1–15. https://doi.org/10.1007/s10508-017-1007-4

Blanchard, R. (2018b). Response to commentaries: Meta-Analysis of probability samples and other new evidence. *Archives of Sexual Behavior*, *47*(1), 49–57. https://doi.org/10.1007/s10508-017-1134-y

Blanchard, R. (2018c). Older brothers and older sisters odds ratios in 36 samples of homosexual males. *Archives of Sexual Behavior*, *47*(4), 829–832. https://doi.org/10.1007/s10508-018-1160-4

Blanchard, R., Barbaree, H. E., Bogaert, A. F., Dickey, R., Klassen, P., Kuban, M. E., & Zucker, K. J. (2000). Fraternal birth order and sexual orientation in pedophiles. *Archives of Sexual Behavior*, *29*, 463–478.

Blanchard, R., Beier, K. M., Gómez Jiménez, F. R., Grundmann, D., Krupp, J., Semenyna, S. W., & Vasey, P. L. (2021). Meta-analyses of fraternal and sororal birth order effects in homosexual pedophiles, hebephiles, and teleiophiles. *Archives of Sexual Behavior*, *50*(3), 779–796. https://doi.org/10.1007/s10508-020-01819-3

Blanchard, R., & Bogaert, A. F. (1996a). Homosexuality in men and number of older brothers. *American Journal of Psychiatry*, *153*(1), 27–31. https://doi.org/10.1176/ajp.153.1.27

Blanchard, R., & Bogaert, A. F. (1996b). Biodemographic comparisons of homosexual and heterosexual men in the kinsey interview data. *Archives of Sexual Behavior*, *25*(6), 551–579. https://doi.org/10.1007/BF02437839

Blanchard, R., & Bogaert, A. F. (1998). Birth order in homosexual versus heterosexual sex offenders against children, pubescents, and adults. *Archives of Sexual Behavior*, *27*(6), 595–603.

Blanchard, R., Cantor, J. M., Bogaert, A. F., Breedlove, S. M., & Ellis, L. (2006). Interaction of fraternal birth order and handedness in the development of male homosexuality. *Hormones and Behavior*, *49*(3), 405–414. https://doi.org/10.1016/j.yhbeh.2005.09.002

Blanchard, R., & Klassen, P. (1997). H-Y antigen and homosexuality in men. *Journal of Theoretical Biology*, *185*(3), 373–378. https://doi.org/10.1006/jtbi.1996.0315

Blanchard, R., Krupp, J., VanderLaan, D. P., Vasey, P. L., & Zucker, K. J. (2020). A method yielding comparable estimates of the fraternal birth order and female fecundity effects in male homosexuality. *Proceedings of the Royal Society B: Biological Sciences*, *287*(1923), 20192907. https://doi.org/10.1098/rspb.2019.2907

Blanchard, R., & Lippa, R. A. (2007). Birth order, sibling sex ratio, handedness, and sexual orientation of male and female participants in a BBC internet research project. *Archives of Sexual Behavior*, *36*(2), 163–176. https://doi.org/10.1007/s10508-006-9159-7

Blanchard, R., & Sheridan, P., M. (1992). Sibship size, sibling sex ratio, birth order, and parental age in homosexual and nonhomosexual gender dysphorics. *The Journal of Nervous and Mental Disease*, *180*(1), 40–47.

Blanchard, R., & VanderLaan, D. P. (2015). Commentary on Kishida and Rahman (2015), including a meta-analysis of relevant studies on fraternal birth order and sexual orientation in men. *Archives of Sexual Behavior*, *44*(5), 1503–1509. https://doi.org/10.1007/s10508-015-0555-8

Blanchard, R., & Zucker, K. J. (1994). Reanalysis of Bell, Weinberg, and Hammersmith's data on birth order, siblings sex ratio, and parental age in homosexual men. *American Journal of Psychiatry*, *151*(9), 1375–1376.

Blanchard, R., Zucker, K. J., Bradley, S. J., & Hume, C. S. (1995). Birth order and sibling sex ratio in homosexual male adolescents and probably prehomosexual feminine boys. *Developmental Psychology*, *31*(1), 22–30.

Blanchard, R., Zucker, K. J., Cohen-Kettenis, P. T., Gooren, L. J. G., & Bailey, J. M. (1996). Birth order and sibling sex ratio in two samples of Dutch gender-dysphoric homosexual males. *Archives of Sexual Behavior*, *25*(5), 495–514. https://doi.org/10.1007/BF02437544

Blanchard, R., Zucker, K. J., Siegelman, M., Dickey, R., & Klassen, P. (1998). The relation of birth order to sexual orientation in men and women. *Journal of Biosocial Science*, *30*(4), 511–519. https://doi.org/10.1017/S0021932098005112

Blitzstein, J. K., & Hwang, J. (2019). *Introduction to probability* (2nd ed.). Taylor & Francis.

Bogaert, A. F., Bezeau, S., Kuban, M., & Blanchard, R. (1997). Pedophilia, sexual orientation, and birth order. *Journal of Abnormal Psychology*, *106*(2), 331–335. https://doi.org/10.1037/0021-843X.106.2.331

Bozkurt, A., Bozkurt, O. H., & Sonmez, I. (2015). Birth order and sibling sex ratio in a population with high fertility: Are turkish male to female transsexuals different? *Archives of Sexual Behavior*, *44*(5), 1331–1337. https://doi.org/10.1007/s10508-014-0425-9

Cantor, J. M., Blanchard, R., Paterson, A. D., & Bogaert, A. F. (2002). How many gay men owe their sexual orientation to fraternal birth order? *Archives of Sexual Behavior*, *31*(1), 63–71. https://doi.org/10.1023/A:1014031201935

Ellis, L., & Blanchard, R. (2001). Birth order, sibling sex ratio, and maternal miscarriages in homosexual and heterosexual men and women. *Personality and Individual Differences*, *30*(4), 543–552. https://doi.org/10.1016/S0191-8869(00)00051-9

Frisch, M., & Hviid, A. (2006). Childhood family correlates of heterosexual and homosexual marriages: A national cohort study of two million Danes. *Archives of Sexual Behavior*, *35*(5), 533–547. https://doi.org/10.1007/s10508-006-9062-2

Gaujoux, R. (2021). doRNG: Generic reproducible parallel backend for 'foreach' loops [Coputer software]. https://CRAN.R-project.org/package=doRNG

Gelman, A., & Loken, E. (2013). The garden of forking paths: Why multiple comparisons can be a problem, even when there is no "Fishing Expedition" or "p-Hacking" and the research hypothesis was posited ahead of time. *Unpublished Manuscript*. http://www.stat.columbia.edu/~gelman/research/unpublished/p_hacking.pdf

Gelman, A., & Stern, H. (2006). The difference between "Significant" and "Not significant" is not itself statistically significant. *The American Statistician*, *60*(4), 328–331. https://doi.org/10.1198/000313006X152649

Grech, V., & Mamo, J. (2020). What is the sex ratio at birth? *Early Human Development*, *140*, Article 104858. https://doi.org/10.1016/j.earlhumdev.2019.104858

Goldfeld, K., & Wujciak-Jens, J. (2021). Simstudy: Simulation of study data [Computer software]. https://CRAN.Rproject.org/package=simstudy

Gómez-Gil, E., Esteva, I., Carrasco, R., Almaraz, M. C., Pasaro, E., Salamero, M., & Guillamon, A. (2011). Birth order and ratio of brothers to sisters in Spanish transsexuals. *Archives of Sexual Behavior*, *40*(3), 505–510. https://doi.org/10.1007/s10508-010-9614-3

Hedges, L. V., & Olkin, I. (1985). *Statistical methods for meta-analysis*. Academic Press.

Jones, M. B., & Blanchard, R. (1998). Birth order and male homosexuality: Extension of Slater's index. *Human Biology*, *70*(4), 775–787.

Khovanova, T. (2020). On the mathematics of the fraternal birth order effect and the genetics of homosexuality. *Archives of Sexual Behavior*, *49*(2), 551–555. https://doi.org/10.1007/s10508-019-01573-1

Microsoft Corporation, & Weston, S. (2021a). *doParallel: Foreach parallel adaptor for the 'parallel' package* [Computer software]. https://CRAN.R-project.org/package=doParallel

Microsoft Corporation, & Weston, S. (2021b). *Foreach: Provides foreach looping construct* [Computer software]. https://CRAN.Rproject.org/package=doRNG

Rafi, Z., & Greenland, S. (2020). Semantic and cognitive tools to aid statistical science: Replace confidence and significance by compatibility and surprise. *BMC Medical Research Methodology*, 244. https://doi.org/10.1186/s12874-020-01105-9

Schagen, S. E. E., Delemarre-van de Waal, H. A., Blanchard, R., & Cohen-Kettenis, P. T. (2012). Sibling sex ratio and birth order in early-onset gender dysphoric adolescents. *Archives of Sexual Behavior*, *41*(3), 541–549. https://doi.org/10.1007/s10508-011-9777-6

Simmons, J. P., Nelson, L. D., & Simonsohn, U. (2011). False-positive psychology: Undisclosed flexibility in data collection and analysis allows presenting anything as significant. *Psychological Science*, *22*(11), 1359–1366. https://doi.org/10.1177/0956797611417632

Skorska, M. N., Coome, L. A., Saokhieo, P., Kaewthip, O., Chariyalertsak, S., & VanderLaan, D. P. (2020). Handedness and birth order among heterosexual men, gay men, and Sao Praphet Song in northern Thailand. *Archives of Sexual Behavior*, *49*, 2431–2448. https://doi.org/10.1007/s10508-020-01774-z

Tran, U. S., Kossmeier, M., & Voracek, M. (2019). Associations of bisexuality and homosexuality with handedness and footedness: A latent variable analysis approach. *Archives of Sexual Behavior*, *48*(5), 1451–1461. https://doi.org/10.1007/s10508-018-1346-9

VanderLaan, D. P., & Vasey, P. L. (2011). Male sexual orientation in independent Samoa: Evidence for fraternal birth order and maternal fecundity effects. *Archives of Sexual Behavior*, *40*(3), 495–503. https://doi.org/10.1007/s10508-009-9576-5

Vasey, P. L., & VanderLaan, D. P. (2007). Birth order and male androphilia in Samoan *Fa'afafine*. *Proceedings of the Royal Society B: Biological Sciences*, *274*(1616), 1437–1442. https://doi.org/10.1098/rspb.2007.0120

Voracek, M., Kossmeier, M., & Tran, U. S. (2019). Which data to meta-analyze, and how?: A specification-curve and multiverse-analysis approach to meta-analysis. *Zeitschrift Für Psychologie.*, *227*(1), 64–82. https://doi.org/10.1027/2151-2604/a000357

Zietsch, B. P. (2018). Reasons for caution about the fraternal birth order effect. *Archives of Sexual Behavior*, *47*(1), 47–48. https://doi.org/10.1007/s10508-017-1086-2

Zucker, K. J., & Blanchard, R. (1994). Reanalysis of Bieber et al.'s 1962 data on sibling sex ratio and birth order in male homosexuals. *The Journal of Nervous and Mental Disease*, *182*, 528–530.

Zucker, K. J., Blanchard, R., Kim, T.-S., Pae, C.-U., & Lee, C. (2007). Birth order and sibling sex ratio in homosexual transsexual South Korean men: Effects of the male-preference stopping rule. *Psychiatry and Clinical Neurosciences*, *61*(5), 529–533. https://doi.org/10.1111/j.1440-1819.2007.01703.x
